# Railway infrastructure maintenance efficiency improvement using deep reinforcement learning integrated with digital twin based on track geometry and component defects

**DOI:** 10.1038/s41598-023-29526-8

**Published:** 2023-02-10

**Authors:** Jessada Sresakoolchai, Sakdirat Kaewunruen

**Affiliations:** Department of Civil Engineering, University of Birmingham, Birmingham, B15 2TT UK

**Keywords:** Civil engineering, Mechanical engineering

## Abstract

Railway maintenance is a complex and complicated task in the railway industry due to the number of its components and relationships. Ineffective railway maintenance results in excess cost, defective railway structure and components, longer possession time, poorer safety, and lower passenger comfort. Of the three main maintenance approaches, predictive maintenance is the trendy one, and is proven that it provides the highest efficiency. However, the implementation of predictive maintenance for the railway industry cannot be done without an efficient tool. Normally, railway maintenance is corrective when some things fail or preventive when maintenance is routine. A novel approach using an integration between deep reinforcement learning and digital twin is proposed in this study to improve the efficiency of railway maintenance which other techniques such as supervised and unsupervised learning cannot provide. In the study, Advantage Actor Critic (A2C) is used to develop a reinforcement learning model and agent to fulfill the need of the study. Real-world field data over four years and 30 km. is obtained and applied for developing the reinforcement learning model. Track geometry parameters, railway component defects, and maintenance activities are used as parameters to develop the reinforcement learning model. Rewards (or penalties) are calculated based on maintenance costs and occurring defects. The new breakthrough exhibits that using reinforcement learning integrated with digital twin can reduce maintenance activities by 21% and reduce the occurring defects by 68%. Novelties of the study are the use of A2C which is faster and provides better results than other traditional techniques such as Deep Q-learning (DQN), each track geometry parameter is considered without combining into a track quality index, filed data are used to develop the reinforcement learning model, and seven independent actions are included in the reinforcement learning model. This study is the world’s first to contribute a new guideline for applying reinforcement learning and digital twins to improve the efficiency of railway maintenance, reduce the number of defects, reduce the maintenance cost, reduce the possession time for railway maintenance, improve the overall safety of the railway operation, and improve the passenger comfort which can be seen from its results.

## Introduction

Demand for railway transportation is increasing every year. Based on the increasing load and speed of rolling stocks, railway maintenance is intensively required due to the rapid deterioration. However, performing railway maintenance is very complicated and is not an easy task. The railway system consists of many components and each component affects the others in different ways. Therefore, railway maintenance is a very complex and complicated task. There is room for the improvement of railway maintenance. Issues that need to be considered are the limited budget which decision-makers have to carefully allocate budget for the optimized maintenance and possession time which will disrupt railway operation. In addition, safety and passenger comfort are also affected by the efficiency of railway maintenance.

Ineffective railway maintenance results in inappropriate resource allocation. Together with the limited budget, railway maintenance efficiency can be worse. For example, track sections with poor conditions may not be maintained while track sections with acceptable conditions are excessively maintained. It can be seen that if the limited resources are allocated properly, the overall railway infrastructure will be able to be maintained in an acceptable range rather than having very good condition and very bad condition track sections. Railway maintenance can be performed in different ways namely corrective maintenance, preventive maintenance, and predictive maintenance (condition-based maintenance). There have been studies proving that corrective maintenance is not appropriate because severities of defects tend to be high as well as their damages and disruptions to normal operation. After all, maintenance is performed when there is something failed so it is unplanned and unscheduled which makes the cost the highest among all kinds of maintenance. In addition, railway operators might be fined because of the interruption of operations. Therefore, corrective maintenance is unpreferable in the present. Preventive maintenance tends to be a better choice because it can prevent defects from occurring so track sections are likely to be free from defects. Moreover, maintenance is performed based on plans so it is much easier to manage. However, the main disadvantage of preventive maintenance is it requires extensive maintenance which causes a significantly high cost to the railway system. Nowadays, predictive maintenance seems to be the most sensible option to perform maintenance activities because it performs as much as necessary to keep railway infrastructure in an acceptable condition. Maintenance is planned based on the current condition of each railway component. Maintenance will be performed only if there are possibilities of failure. Together with powerful computing hardware, the use of predictive maintenance becomes more possible when machine learning is applied. Machine learning is used for railway maintenance for different purposes such as defect prediction^1^ or track circuit maintenance^2^. The accuracy of the prediction can be high as 80%^1^ which demonstrates the potential of machine learning. However, problems in the railway industry are complicated and complex. Deep learning seems to be able to achieve better performance than traditional machine learning because it can better caption the non-linear characteristics of the railway industry.

Deep reinforcement learning is used to solve many problems nowadays. However, use in railway maintenance is still limited. An agent in the reinforcement learning model can learn through model training to maximize rewards or minimize penalties. In this case, maintenance costs and defects can be considered penalties that the agent has to minimize.

The aim of the study is to develop a reinforcement learning model integrated with digital twins to improve the efficiency of railway infrastructure maintenance based on track geometry parameters and track component defects. data collection is done by obtaining filed data from 2016 to 2019 when the length of the track is 30 km. Data from defect inspection and maintenance records are also used to develop the reinforcement learning model. In total, there are more than 300 k samples that are used to train the reinforcement learning agent. The machine learning technique is Advantage Actor Critic (A2C) which is proven that the performance is better than the traditional techniques such as Deep Q-learning (DQN) while the processing time is shorter. The digital twin is applied as the data management platform for long-term implementation because it can store data and historical data can be tracked. Some benefits of integration between machine learning and digital twins are it can improve the efficiency of data management and productivity because all data can be stored in a single model and data can be used to make decisions during different stages of the project. The accuracy of prediction is better because the information is extracted from the digital twin model directly which can remove human errors. The digital twin model also improves collaboration because the digital twin model can be shared with the relevant parties and responsible persons. Last but not least, the integration between the digital twin and machine learning will improve cost efficiency and sustainability because the digital twin has been proven that it can save the cost of the project so the required resource for activities in the project will be reduced which is sustainable. The contributions of the study are it can be used as a guideline for railway operators to improve railway maintenance efficiency and it also has the potential to provide predictive maintenance plans which can reduce the number of defects, maintenance cost, and possession time for railway maintenance. Last but not least, the approach presented in the study can improve the safety and passenger comfort of the railway system. The study's novelties include the use of A2C, which is faster and produces better results than other conventional techniques, the consideration of each track geometry parameter separately rather than in combination to create a track quality index (TQI), and the development of the reinforcement learning model using field data. Moreover, it is worth noting that supervised and unsupervised learning cannot solve the problem mentioned in this study because the prediction from supervised and unsupervised learning is done one time only. Different from those two, reinforcement learning can use historical data and action to respond continuously along the stages of learning. This is one of the main contributions and characteristics that other machine learning techniques do not have.

## Applications of reinforcement learning in the railway system and other transportation systems

There are different methods that can be used to plan railway maintenance. Sedghi et al.^3^ have performed a literature review on this topic. Most of the techniques from their review were based on mathematical approaches or probability approaches such as stochastic mode, mixed integer programming, simulation, Markovian model, and machine learning which tend to attract attention nowadays and also provide satisfying performance. The following are some studies related to reinforcement learning or its fundamentals.

Lopes Gerum et al.^1^ proposed an approach for predictive maintenance by using a non-linear regression model to predict defects that might take place in track sections. Techniques that they used to develop predictive models of defects were random forest (RF) and recurrent neural network (RNN). They found that RNN provided a better accuracy of 80% while RF resulted in an accuracy of about 77%. The predictive models were used to classify the severity of defects into two levels which were yellow (minor defects) and red (major defects). Then, they applied discounted Markov decision process to prepare inspection and maintenance schedules. They found that they could save on the maintenance cost using the proposed approach. In that study, the long-term master policy and maintenance were developed using the proposed approach however detailed maintenance schedule needed to be studied further.

Consilvio et al.^2^ applied a similar technique to apply machine learning with railway earthwork and track circuit maintenance. They applied k-mean and a support vector machine to generate numerical data. Then, they applied Monte Carlo simulations and mixed integer linear programming mathematical models for optimization. They evaluated the performance of the approach by using key performance indicators (KPIs). They found that the proposed approach could save maintenance costs and be applied for other purposes. However, the main limitation of their study is all data were generated and tested in a laboratory environment. Therefore, practice in real-world situations needs to be proven.

From the literature review, the use of reinforcement learning in railway maintenance has not been widespread due to the limited number of related studies. There is only one study that has been done by Mohammadi and He^4^. They applied Deep Q-learning (DQN) to develop a decision-making tool for railway maintenance. Inputs of this approach were the track quality index (TQI) and hazard index. They obtained these two indicators by using RF. Then, they used these two to train the reinforcement learning model. Their action spaces consisted of five activities which are preventive tamping, preventive grinding, condition-based tamping, condition-based grinding, and renewal. They found that applying the developed approach could reduce the TQI and hazard index when compared to the baseline.

Although the application of reinforcement learning in railway maintenance is limited, reinforcement has been applied in different aspects. Šemrov et al.^5^ applied reinforcement learning for rescheduling the single-track railway. They applied Q-learning to develop a machine learning model and found that they could improve railway scheduling. Other studies applied reinforcement learning for railway scheduling such as^6–11^ which they all found that reinforcement learning could improve the efficiency of railway scheduling. Other applications of reinforcement learning for railway systems are pantograph control^12^, resource allocation^13^, speed regulation^14,15^, power and energy management^16–18^, design^12^, and defect detection^19^. All studies have found that reinforcement learning can solve or improve a specific problem in each study.

In terms of maintenance, reinforcement learning has been applied in other industries. Rocchetta et al.^20^ applied Q-learning to optimize the maintenance of power grids. They found that all corrective maintenance could be gotten rid of. Yao et al.^21^ applied DQN to plan a long-term maintenance plan for pavement. They found that they could reduce the number of maintenance activities by 50% by using reinforcement learning. Xanthopoulos et al.^22^ applied DQN for manufacturing system maintenance. They found that they could reduce the maintenance cost by 10% approximately which was also found by Paraschos et al.^23^.

From the literature review, it can be seen that the use of reinforcement learning for railway infrastructure maintenance is in the developing process and there have not many studies have been done. Only one study seems to be related^4^. Moreover, there are gaps in studies on this topic. For example, each track geometry parameter can be considered for more practical, comprehensive maintenance activities that can be included in the reinforcement learning model, field data can be combined with the reinforcement learning model, or a digital twin can be integrated with the reinforcement learning model for asset management and more efficiency. Therefore, this study aims to fulfill these gaps as much as possible by developing an approach to integrated digital twin and reinforcement learning for improving the efficiency of railway infrastructure maintenance based on an individual track geometry parameter and track component defect. Detailed maintenance activities and filed data are included in the reinforcement learning model development to ensure that the developed model imitates the real-world situation as much as possible. Expected outcomes from the approach are the maintenance cost of railway infrastructure and occurring defects are reduced which represents better efficiency of maintenance.

## Methodology

### Reinforcement learning and advantage actor critic (A2C)

**Figure 1:**
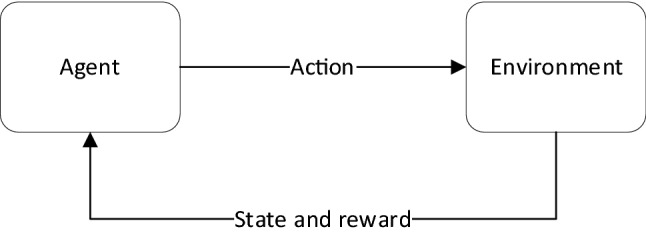
Simple process of reinforcement learning.

Reinforcement learning is one of three main categories of machine learning among supervised and unsupervised learning. In the present, reinforcement learning gains a lot of attraction due to its capabilities. In reinforcement learning models, agents are trained and learn from environments^24^. Environments have rules for agents to follow such as constraints and available actions. Agents will interact with environments depending on the states (${s}_{t}\in S$ where $S$ is possible states) which can be a discrete time step ($t=1, 2, 3, \dots , n$) by performing actions (${a}_{t}\in a\left({s}_{t}\right)$ where $a\left({s}_{t}\right)$ is possible actions in state ${s}_{t}$). After that, agents will get rewards (or penalties ${R}_{t}$) for performing actions^4^. Then, states will change to the next states and agents will perform actions again. This process will repeat until the end of training or environments’ states. The purpose of agent training is to maximize rewards or minimize penalties. A simple diagram can be shown in Fig. 1.

From the overview of reinforcement learning, it can be seen that supervised and unsupervised learning cannot learn and solve described problems. This is because supervised and unsupervised learning are trained by loss functions but reinforcement learning agents are trained by considering the rewards of each state (short-term rewards), total rewards at the last state (long-term rewards), or both through value functions. Value functions identify how good agents perform in each state.

Based on policies, reinforcement learning can be categorized into two main groups which are on-policy and off-policy reinforcement learning. For off-policy, an updated policy is different from a behavior policy^25^. Agents are greedy and estimate rewards for the future. For on-policy, agents estimate the values of followed policy and an updated policy is the same as a behavior policy. On-policy reinforcement learning is suitable when the reinforcement learning model aims to optimize processes or decision-making. Off-policy reinforcement learning is suitable when agents need to explore new ways to maximize rewards such as paintings or drawings that agents need to generate new data. Examples of off-policy reinforcement learning are Q-learning, Deep Deterministic Policy Gradients (DDPG), and Behavioral Cloning^26^. Examples of on-policy reinforcement learning are Advantage Actor-critic (A2C) and Proximal Policy Optimization (PPO). It can be seen that on-policy reinforcement learning is more suitable for the problem in this study because the aim is to improve maintenance efficiency. Moreover, it has been proven that A2C performed better than DQN by 20% while the training time is shorter^27^. Therefore, this study will apply A2C to developing the reinforcement learning model.

A2C is a hybrid of two types of reinforcement learning techniques which are policy-based and value-based to obtain the advantages of both. By their names, the policy-based technique allows agents to learn policies (or actions) from input states before taking action(s) while the value-based agents use the predicted value of states or actions to take action(s). in other words, A2C is a combination between Q-learning and Policy Gradient (PG). A2C comprises two main parts (neural networks) which are the actor and the critic. The actor part is used to select actions in each state using PG and the critic part is used to consider the value of that state using Q-learning to calculate a policy function ${\pi }_{\theta }\left({s}_{t},{a}_{t}\right)$. The critic part will evaluate whether states are good or bad then the actor will learn and teach agents to take actions that lead to good states based on this insight via an advantage function $A\left({s}_{t},{a}_{t}\right)$ which calculated from a value function ${\widehat{q}}_{w}\left({s}_{t},{a}_{t}\right)$ and an average value of a state $V\left({s}_{t}\right)$ as Eq. (1). From the equation, a positive $A$ is preferable because it means that the training is in the correct direction.

1 $$A\left( {s_{t} ,a_{t} } \right) = \hat{q}_{w} \left( {s_{t} ,a_{t} } \right) - V\left( {s_{t} } \right)$$

In each state $s_{t}$, information is passed through the actor and critic. Then, an agent is trained to take action(s) ${a}_{t}$ which will be input for the critic. ${s}_{t}$ and ${a}_{t}$ are used by the critic to calculate the Q-value ${\widehat{q}}_{w}\left({s}_{t},{a}_{t}\right)$ and the actor will update the policy based on this value as well as the critic. Therefore, both critic and actor will learn together along the training process. After ${a}_{t}$ is performed, new state ${s}_{t+1}$ and rewards ${R}_{t+1}$ are calculated. This process will repeat as the process of reinforcement learning mentioned above. By the concept, A2C has a more comprehensive capability than Q-learning and DQN when performance variation is less due to PG. This is because policy-based techniques use all states to calculate rewards so the performance can be variant.

### Data Characteristics and preliminary analysis

**Figure 2:**
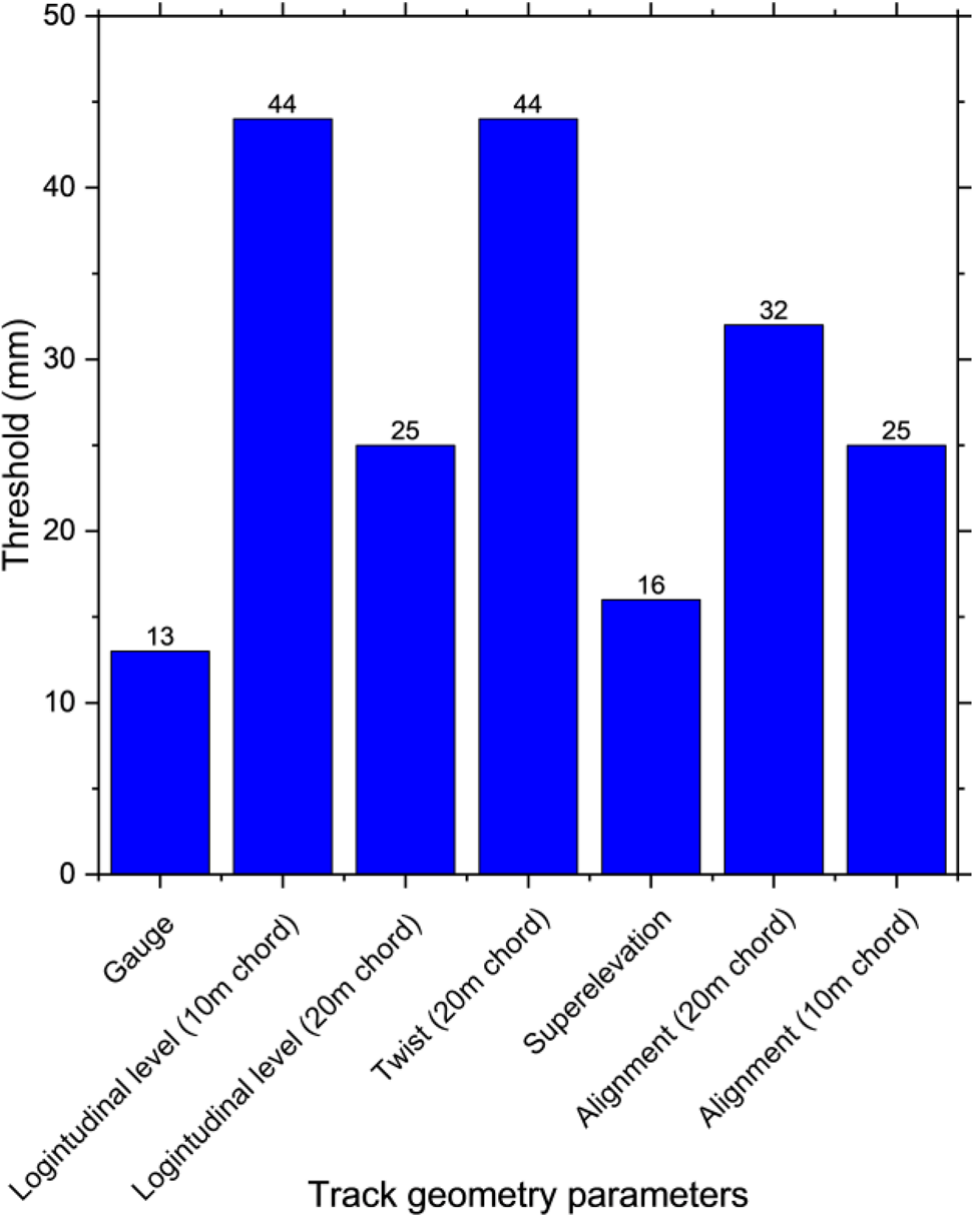
Threshold of each track geometry parameter.

Data used in the study are obtained from a 30-km railway section during 2016–2019 from MRS Logística S.A. The data are from many sources. First, track geometry parameters are obtained using track geometry cars which collect foot-by-foot track geometry parameters. This part of the data consists of locations of measurement, superelevation, longitudinal level (10 m chord), longitudinal level (20 m chord), alignment (10 m chord), alignment (20 m chord), gauge, and twist (20 m chord). In fact, track geometry cars also collect other data but only relevant data are focused on. In total, there are more than 300 k sections that can be used as the input of the reinforcement learning model. Locations of measurement will be used as references for comparison. Therefore, there are seven track geometry parameters that will be used as inputs of the reinforcement learning model. In other words, they will be used as stated for the agent to select the next actions. From the organization, thresholds are defined as four priorities. Priority 1 means the track geometry parameters are very bad and track sections need to be maintained as soon as possible while priority 4 means track sections need to be included in the regular maintenance plan. In this case, priority 4 which is the less concerned trigger level will be used as the threshold because this study aims to perform maintenance activities that keep track free from defects or keep them in the minimum numbers. The threshold of each track geometry parameter is shown in Fig. 2. If any track sections have track geometry parameters that exceed the threshold, those sections are considered defective sections, and the number of defects is based on the number of exceeding track geometry parameters.

**Figure 3:**
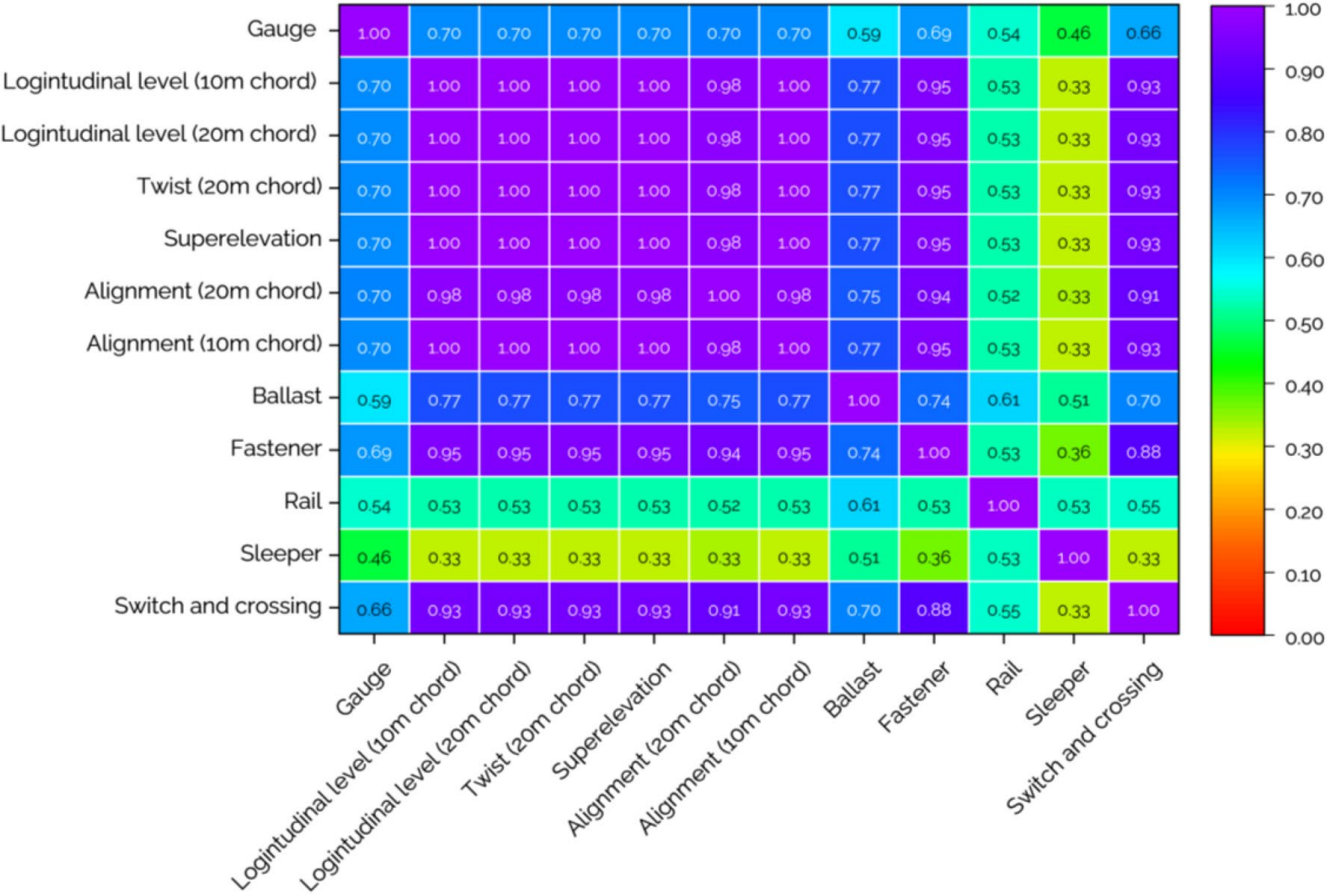
Correlation between defects.

The next source of data is defect inspection reports collecting track component defects. The report informs the dates and locations of each defect finding. There are 71 different types of track component defects. However, to simplify the data, they are grouped into five categories based on track components which are ballast, fastener, rail, sleeper, and switch and crossing. Different from track geometry defects, the consideration of track component defect occurrence is simpler. If defects are found, those track sections are considered defective and the number of defects is based on the number of track component defects. Combining track geometry and component defects, the total number of defect categories is 12, seven from track geometry defects and five from track component defects. These 12 types of defects will be used as states in the reinforcement learning model to train the agent to perform maintenance activities when the target is keeping every track section free from defects. From the preliminary analysis, correlation analysis is conducted. Correlations between defects both track geometry and component defects are shown in Fig. 3. As mentioned, the consideration of track component defects is simple. They are considered defective when they occur. For track geometry defects, track geometry parameters are compared with thresholds as shown in Fig. 2. If parameters exceed the threshold, they are considered defective. From Fig. 3, it can be seen that every track geometry defect is highly correlated except gauge which has a relatively low correlation with other track geometry defects. It means if a track section has some track geometry defect, except gauge, it tends to have other defects as well. For track component defects, the correlation seems to be lower compared to track geometry defects. that means track component defects seem to be more independent. However, it is worth noting that switch and crossing and fastener defects have a high correlation with track geometry defects when the correlation is more than 0.90. This insight will be beneficial for railway operators to investigate track defects and plan maintenance. From the analysis of track geometry records and defect inspection reports, the average number of defects in track sections is two. In this case, there are twelve categories of the defect so two defects on average represent that the efficiency of maintenance should be improved.

The last source of data is maintenance records in the same period of interesting duration which is 2016–2019. From the maintenance records, there are seven maintenance activities consisting of tamping, rail grinding, ballast cleaning, sleeper replacement, rail replacement, fastening components replacement, and ballast unloading. These seven maintenance activities will be the action space for the reinforcement learning model. In this case, the action space can be considered as seven binary spaces in which each maintenance activity can be done or not done. However, in reality, the performing of maintenance activities is more complicated because they can be combined from none to seven activities. Therefore, to consider the possible actions in each state of the reinforcement learning model, the principle of combination is applied. The combination is calculated using Eq. (2) where $n$ is the number of options and $r$ is the size of combination. From the equation, $n$ equals to seven and $r$ can be varied from zero to seven. The summation of the combinations or possible actions will be 128. From the maintenance records, there are more than one million sets of data that can be considered that it is comprehensive enough to compare maintenance activities, changes of track geometry parameters, and occurrence of track component defects in forms of normal distribution.

2 $$Combinations = \frac{n!}{{\left( {n - r} \right)!r!}}$$

**Figure 4:**
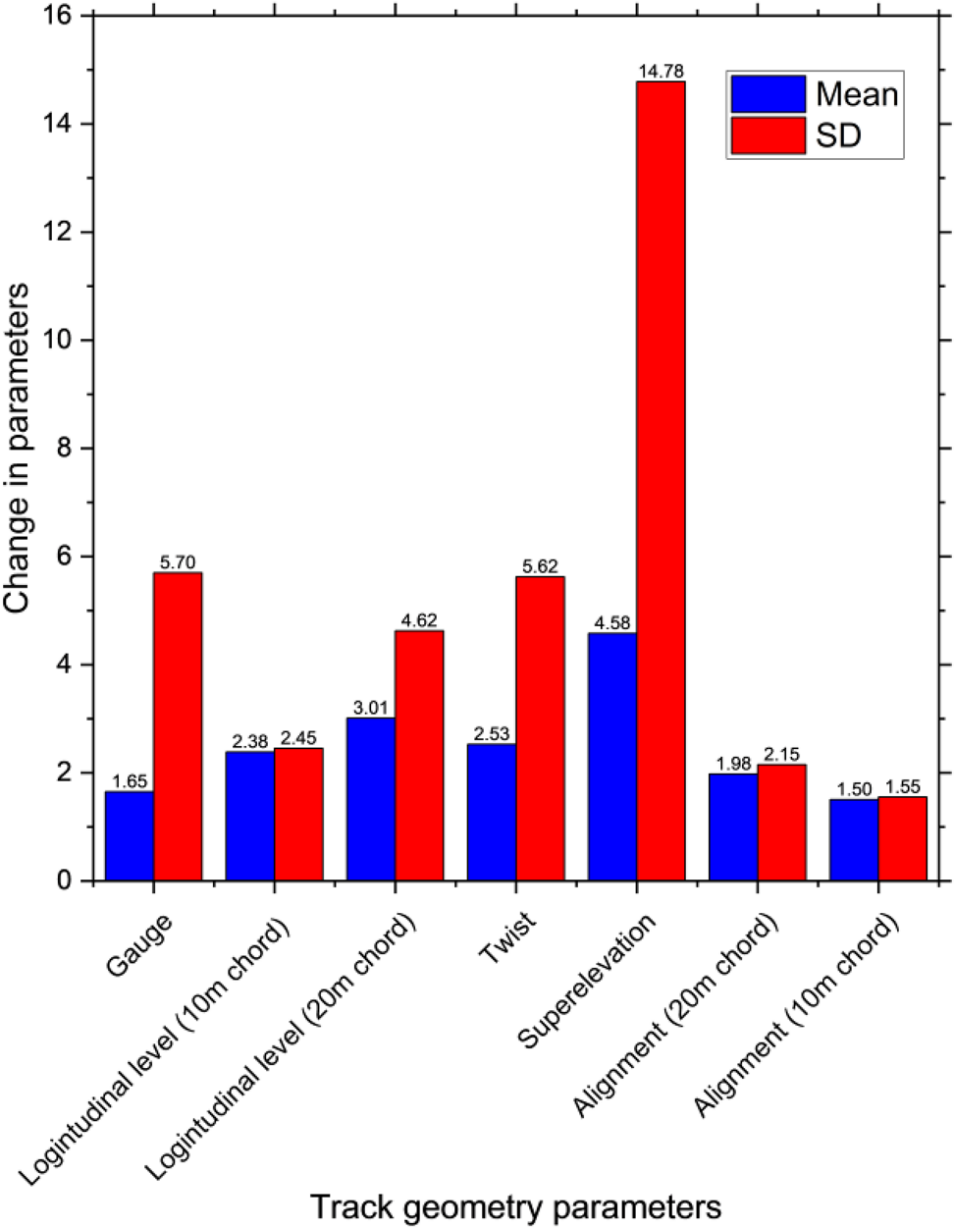
Track geometry improvement when tamping is performed.

**Figure 5:**
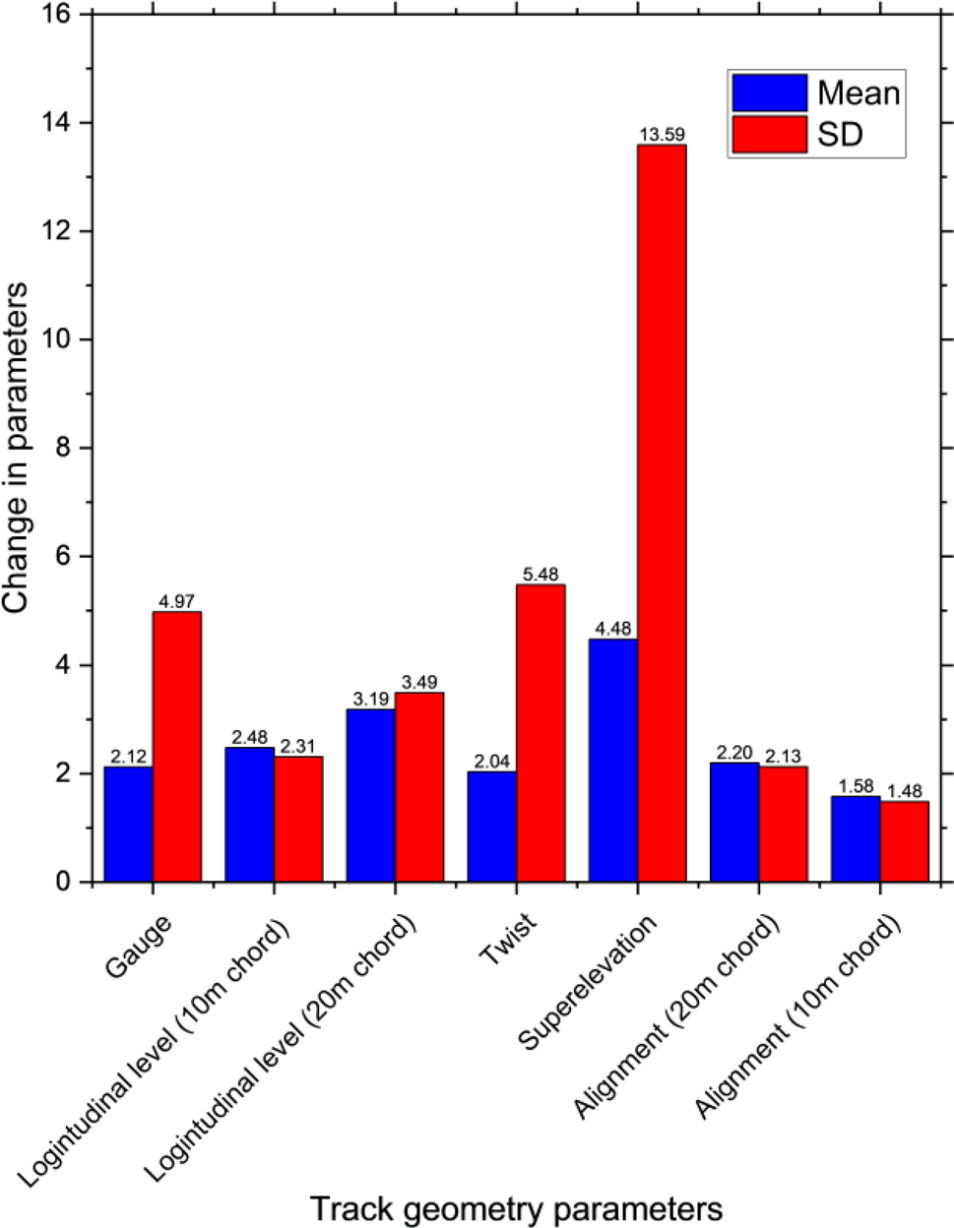
Track geometry deterioration when tamping is not performed.

**Figure 6:**
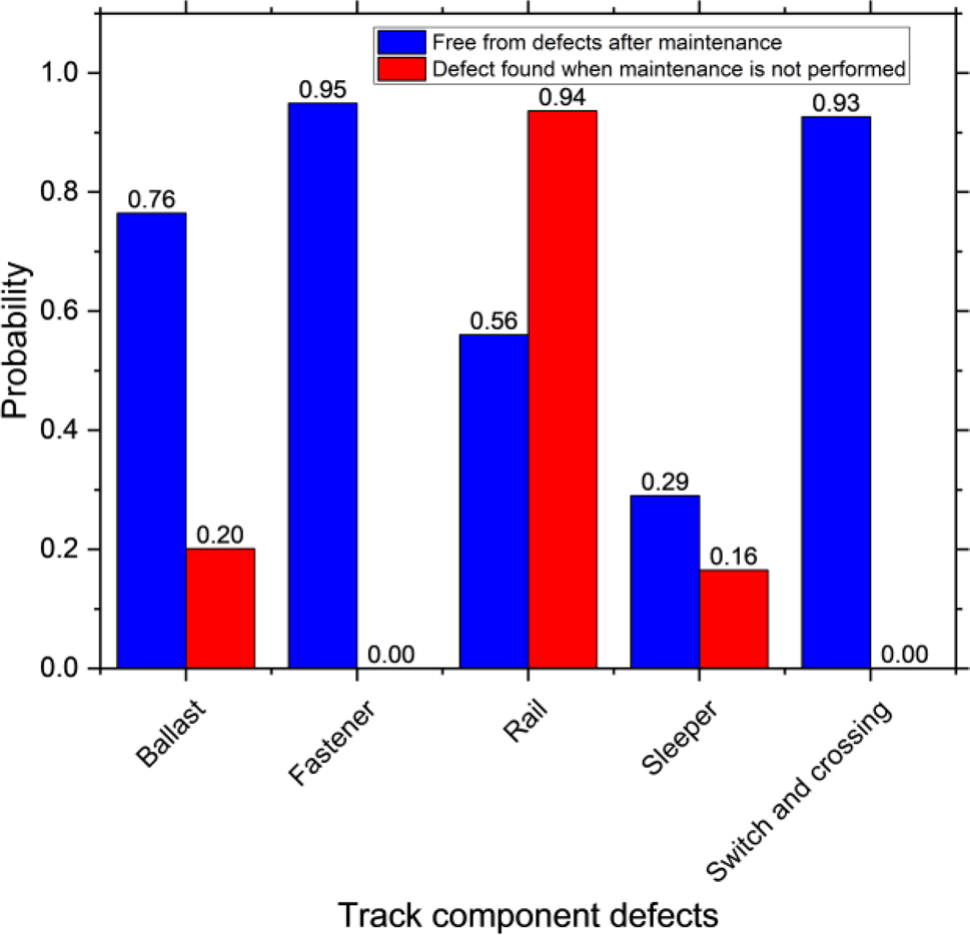
Relationship between defect occurrence and rail grinding.

From the analysis, examples of improvements and deterioration in track geometry parameters related to tamping in the aspect of a normal distribution are shown in Figs. 4 and 5 respectively. Mean and standard deviation (SD) are used as representatives. From the figures, they are only two simple cases that are used to analyze. There are also 126 more cases based on the possible actions that will be used to develop the reinforcement learning model. Also, track component defects, an example of probability when component defects are not found and found when rail grinding is performed and not performed respectively is shown in Fig. 6. As track geometry parameters, the figure shows a simple case only. In fact, there are complicated combinations of maintenance activities that affect the track component defect occurrence that is considered and used as inputs of the reinforcement learning model. From preliminary analysis, it is found that maintenance efficiency is not very good. Some track sections are in good condition but excessive maintenance activities are conducted. On the other hand, some track sections are in bad condition or have many kinds of defects however there are no maintenance activities conducted in those sections. As a result, the conditions of those sections are much worse in the following year with rapid deterioration.

At this stage, all required data is ready for developing the reinforcement learning model. Data will be used as inputs for the reinforcement learning environment which is a rule of the reinforcement learning model. The most challenging issue is how to update the states when the agent takes action. In this case, the states consist of twelve sub-states which are track geometry parameters and occurrences of track component defects. In terms of track geometry parameters, states are real numbers indicating the size of irregularities. In terms of track component defects, states are binary numbers indicating whether there is each defect. To update the states of the reinforcement learning model, changes in each state are considered using field data from track geometry measurements, defect inspection reports, and maintenance records. Changes of states are based on these data using normal distribution related to specific performed maintenance activities as mentioned. Now, all required data are ready for developing the environment for the reinforcement learning model.

### Problem description

To summarize components of reinforcement learning, there are mainly five components which are agent, environment, states, actions, and rewards. The agent interacts with the environment by taking action and obtaining rewards. The environment is everything outside excluding the agent. In addition, the environment defines rules and the nature of the reinforcement model. States are timesteps of the environment or information provided to the agent. Actions are how the agent interacts with the environment. Actions can be discrete or continuous depending on the environment. Rewards are scalar values that the agent obtains from the environment. It represents the success of the agent.

**Figure 7:**
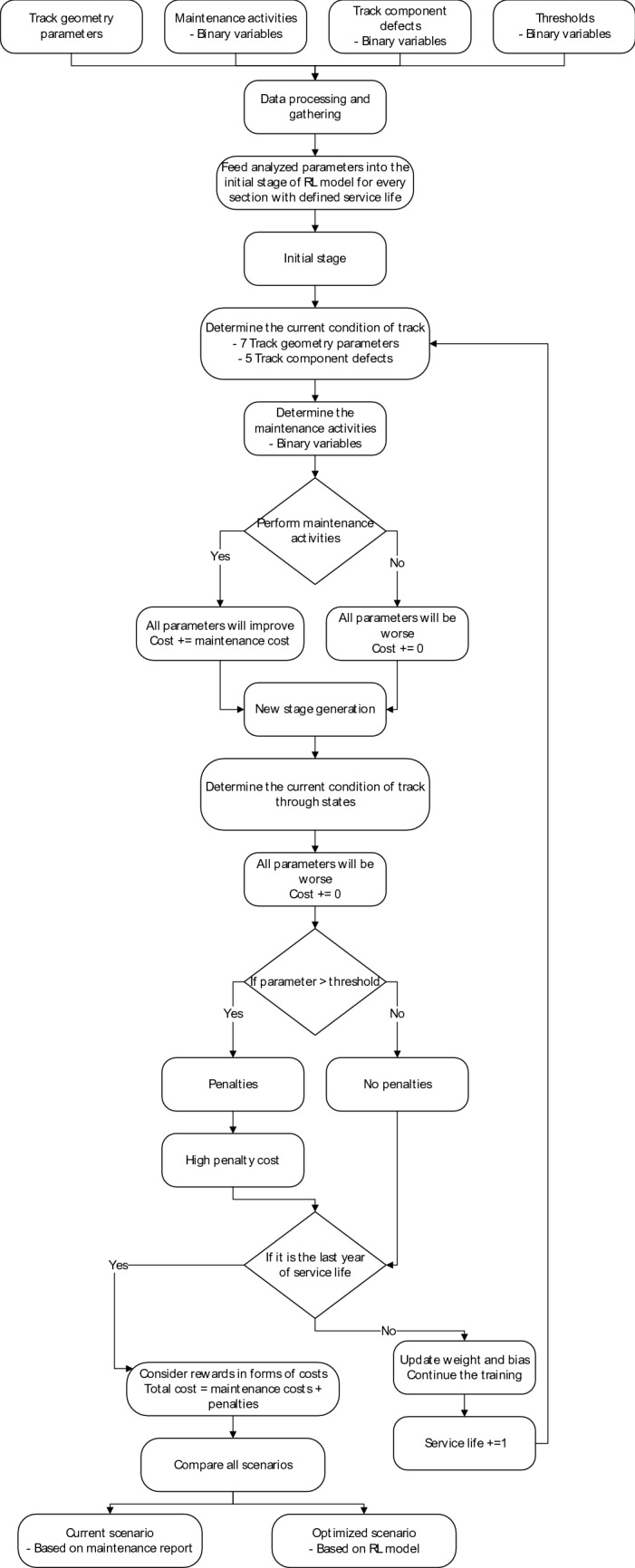
Workflow of the reinforcement learning model.

In the study, the agent is trained to take action by performing maintenance activities consisting of seven activities which are tamping, rail grinding, ballast cleaning, sleeper replacement, rail replacement, fastening components replacement, and ballast unloading as mentioned. There are 128 possible combinations of maintenance activities that the agent can choose to take action on. Each action results in the improvement of track geometry parameters and probabilities of track component defect occurrence. There are twelve states in the environment which are seven track geometry parameters and five track component defect occurrences. They are superelevation, longitudinal level (10 m chord), longitudinal level (20 m chord), alignment (10 m chord), alignment (20 m chord), gauge, twist (20 m chord), ballast, fastener, rail, sleeper, and switch and crossing. In the first state, every parameter is defined according to the field data. Then, the agent has to take action by choosing which maintenance activities it will perform. After taking action, the environment will react by generating a set of new states considering the proper values of each state based on the field data. This process will repeat until the end of the training. In this case, the number of states is set to 100. From this condition, the environment is developed based on the condition of this study to make sure that it will be suitable for real-world application. Rewards are defined in two categories which are rewards in terms of maintenance costs and penalties when there are defects. Track geometry defects are based on Fig. 2 while track component defects are based on the occurrence as mentioned. In this study, the penalties for defect occurrence are set relatively high compared to the maintenance cost because agent training aims to minimize the cost and reduce the number of defects. When the training is done, performance will be evaluated by comparing the number of performed maintenance activities and the number of defects. Two scenarios will be compared which are the data based on the field data and results from the reinforcement learning model. Moreover, loss and policy entropy is used to present the performance of the reinforcement learning model as well. The loss represents the defect occurring in the track section and policy entropy presents how well the agent responds to problems. The targeted values of these two parameters are 0. To summarize the overall process of the developed reinforcement learning model, the chart is shown in Fig. 7.

To ensure the performance of the reinforcement learning model, hyperparameter tuning is conducted including the sensitivity analysis to demonstrate the effects of each hyperparameter. The focused hyperparameters are learning rate, epsilon, and discount factor. The learning rate represents how far the agent goes to another timestep. In the study, the timestep or epoch is set to 100,000 or the agent will be trained 100,000 times. Epsilon represents the proportion between exploration and exploitation. This hyperparameter is used to force the agent to explore new approaches to improve the policy or how to take action under different situations. Last, the discount factor identifies how much the agent cares about rewards in the future. In some cases, the agent may prioritize short-term rewards rather than the overall rewards in the final states. Therefore, to prevent this, the discount factor is used to regulate the agent. The ranges of the learning rate, epsilon, and discount factor tuning are 0 to 1, 1e−9 to 1e−0, and 0.09 to 0.99 respectively.

### Digital twin

**Figure 8:**
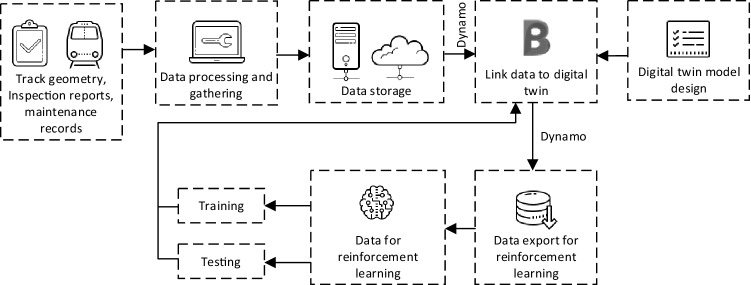
Digital twin and reinforcement learning model integration.

To integrate the machine learning model with a digital twin, the framework is based on^28^ which a detailed process was explained. The summarized flowchart is shown in Fig. 8. Data collection is done by obtaining required data from track geometry measurements, defect inspection reports, and maintenance records. Then, all data are processed to be ready for the reinforcement learning model as described in the previous section. In this stage, data can be fed into the reinforcement learning model directly. However, to develop a data management platform using digital twin, data will be stored in a defined system such as a digital twin model, server, or cloud system for backup. In addition, some parts of data can be called through the digital twin model via hyperlinks in case those data are not appropriate to be stored in the digital twin model such as data with big size or long sequences. To store data in the digital twin model, the model has to be designed to support the data aimed to be stored. Now, the digital twin model has the capability to store data or information. Data exchange can be done using a data management platform. In this case, the digital twin model is developed using Autodesk Civil3D which has all the required capabilities. Moreover, the software also provided Dynamo which built-in add-on that has a function to achieve data management. Therefore, this set of software is used to develop the digital twin model. Data import and export are done as described. Then, data are processed and fed into the reinforcement learning model using the approach mentioned in the previous section. The developed digital twin model has the ability to develop into a 6D model or nD model depending on data wanted to save in the digital twin model such as cost, schedule, carbon emission, or other data.

## Result and discussion

### Reinforcement learning

**Figure 9:**
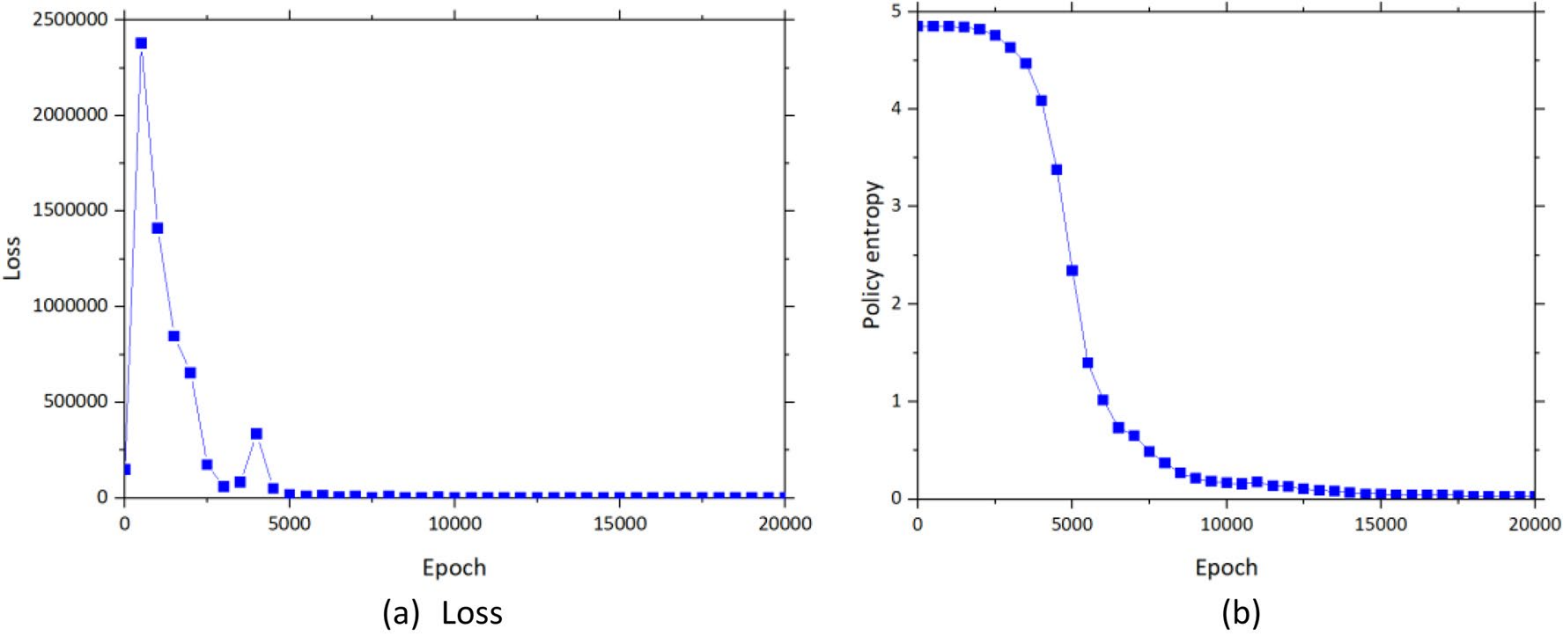
Reinforcement learning model performance (**a**) loss and (**b**) policy entropy.

From the reinforming learning model training, the number of epochs is set to 100,000. Different combinations of hyperparameters are tried to find the best reinforcement learning model. The reinforcement learning model is developed based on Python. The reinforcement learning technique used in the study is A2C. Results in terms of loss and policy entropy are shown in Fig. 9a,b respectively. It is noted that although the epoch is set to 100,000, the figures present the epoch until 20,000 to make the figures can be observed clearly. It can be seen that the training is converged after the reinforcement learning model is trained for 20,000 epochs. As mentioned, the targeted values of loss and policy entropy are 0. This indicates that the reinforcement learning model can find the best policy to deal with the problem in the study. The best combination of the hyperparameters from tuning is a learning rate of 0.007, an epsilon of 1e−5, and a discount factor of 0.99.

**Figure 10:**
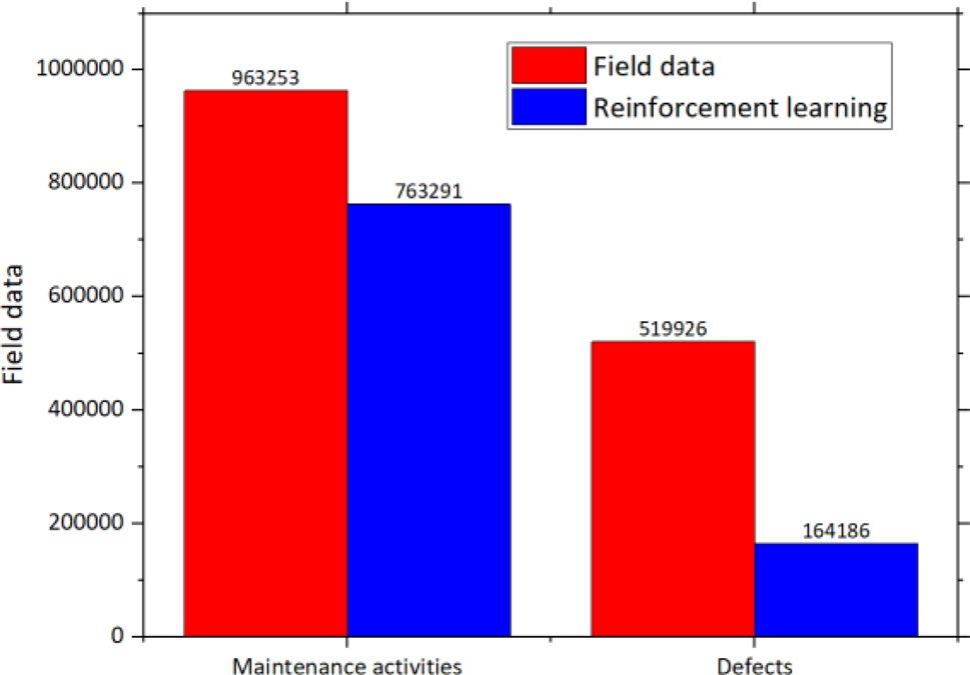
The number of maintenance activities and defects from field data and reinforcement learning model.

**Figure 11:**
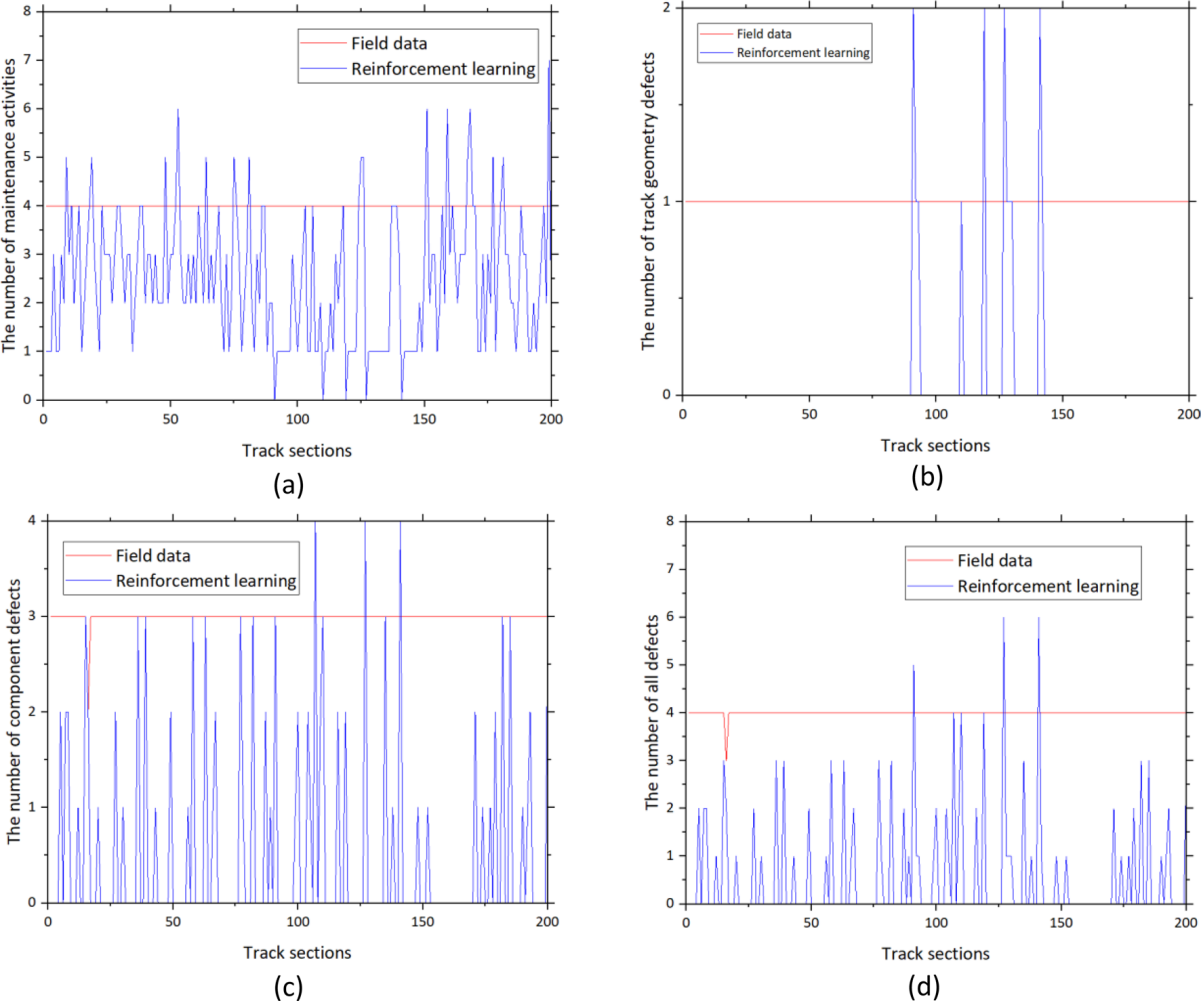
Examples of the maintenance efficiency improvement based on track sections (**a**) maintenance activities, (**b**) track geometry defects, (**c**) track component defects, and (**d**) all defects.

The number of performed maintenance activities and defects from the field data compared to the results from the reinforcement learning model is shown in Fig. 10. It can be seen that the number of both maintenance activities and defects significantly decreased. In the case of maintenance activities, the number decreases from 963 to 763 k or 20.76%. In the case of defects, the number decreases from 520 to 164 k or 68.42%. The number of defects significantly decreases although the number of maintenance activities decreases by 20% approximately. This conforms to the result from the preliminary analysis that maintenance has not been conducted efficiently. Some track sections need maintenance while some need relatively less maintenance. When the maintenance is done properly, the efficiency can be improved a lot. It can be observed from the significant reduction in defects or 68% while the required maintenance reduces by 20% only. On average, the number of performed maintenance activities of field data is 3.4 per section while the average number from the reinforcement learning model is 2.7 per section. The average number of defects from the field data is 2 per section while the number from the reinforcement learning is 0.5 per section. Examples of the comparison between the number of maintenance activities and defects based on track sections are shown in Fig. 11. In the chart, the blue line represents the results from the reinforcement learning model while the red line represents the result from the field data. From Fig. 11a, the number of performed maintenance activities is four for all sections which can be inferred that the maintenance activities are performed based on the plan without considering the exact condition of track sections. However, the results from the reinforcement learning model can better respond to the current conditions of track sections. This can reduce unnecessary maintenance activities which will also save maintenance costs and time. At the same time, the number of track geometry and track component defects is reduced significantly according to Fig. 11b–d. Track component defects are more common than track geometry defects based on Fig. 11b,c. The use of planned maintenance from the reinforcement learning model can almost remove all track geometry defects as shown in Fig. 11b. From Fig. 11d, the average number of overall defects from the field data is four while the number of defects from the reinforcement learning model is significantly lower. However, it is worth noting that the number of defects from the reinforcement learning model is higher than the field data sometimes. This is because of the stochastic behavior of the railway system which is complicated and defects can take place sometimes although the tracks are well maintained.

From the figure, examples of 200 track sections are presented. It can be seen that the overall number of maintenance activities and defects is lower in the case of the reinforcement learning model. However, the number from the reinforcement learning model is higher than the field data due to the stochastic characteristic of the railway system. Although some track sections are maintained, defects still occur which is challenging in the railway aspect.

**Figure 12:**
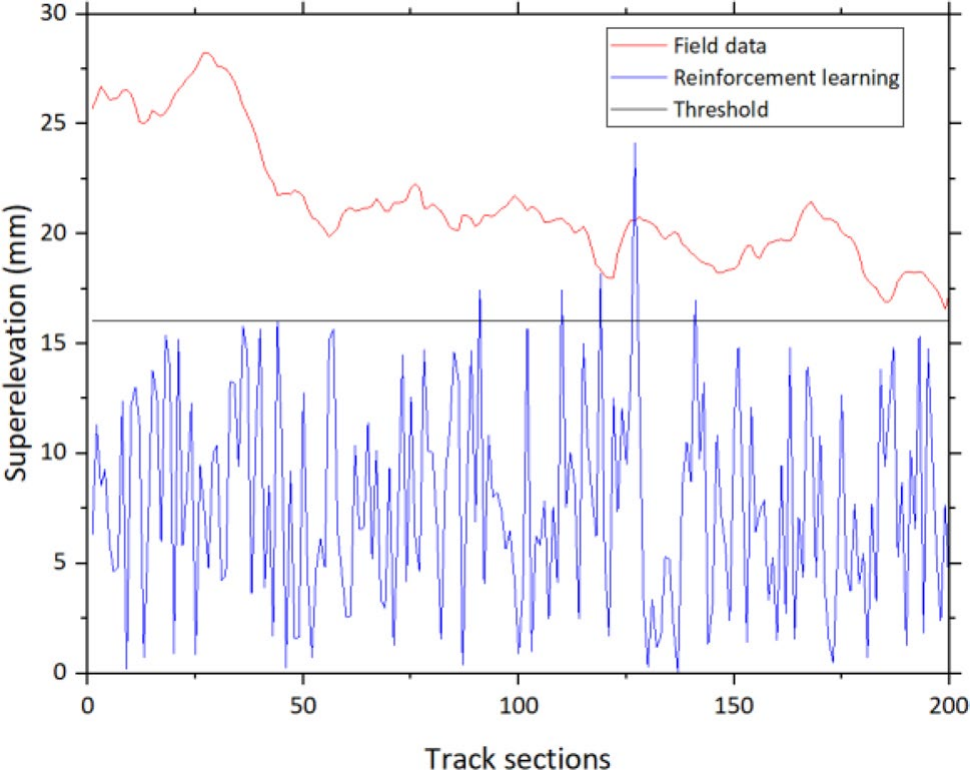
Examples of superelevation.

To observe defects in detail, superelevation is used as an example. From Fig. 12, the threshold of superelevation is 16 mm. From the field data, all values from the first 200 sections exceed the threshold which is unsatisfying. However, results from the reinforcement learning model show that most of the superelevation is in the acceptable range although there is some minority of sections where the values exceed the threshold which is unavoidable.

**Figure 13:**
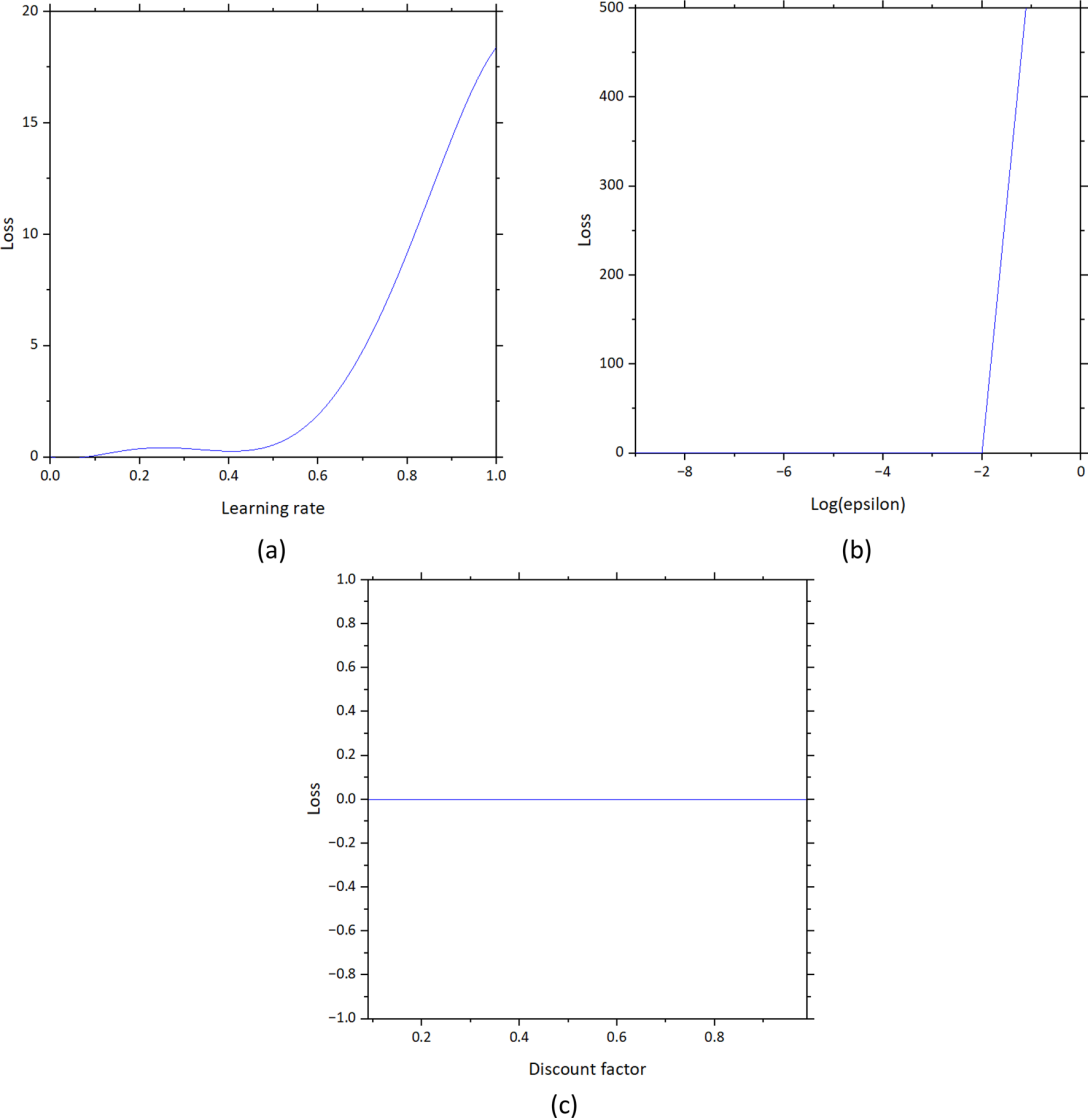
Results from sensitivity analysis and hyperparameter tuning (**a**) learning rate, (**b**) log(epsilon), and (**c**) discount factor.

For the sensitivity analysis and hyperparameter tuning, there are three interesting hyperparameters as mentioned which are learning rate, epsilon, and discount factor. The results are shown in Fig. 13. It can be seen that the smaller learning rate results in better performance of the reinforcement learning model. when the learning rate is higher than 0.5, the loss tends to increase rapidly. This characteristic is also found in epsilon. When the log(epsilon) is higher than − 2 or epsilon higher than 0.01, the loss also rapidly increases. However, this not happens in the case of the discount factor. From the figure, it can be seen that the discount factor does not have any effect on loss in this study.

### Digital twin

**Figure 14:**
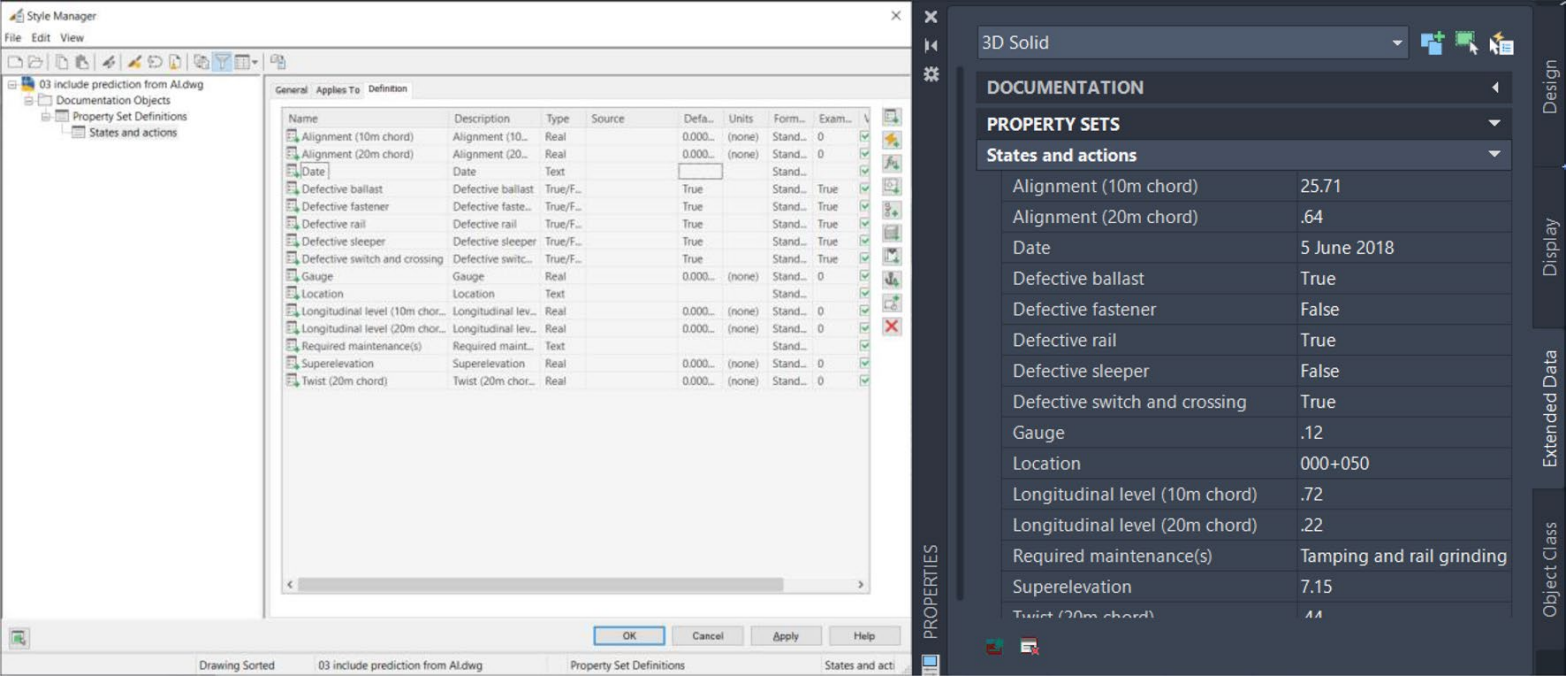
States and actions defined in the digital twin model.

To integrate reinforcement learning with a digital twin, the digital twin model is developed using the sequences mentioned in the methodology. The states and actions for the reinforcement learning model are stored in the digital twin model via the property set definitions. States and actions can be defined in the property set definitions according to their types such as real, integer, or true–false for binary states (defect found or not found/perform or not perform maintenance). In addition, the locations of track sections and the date of inspection and maintenance can be stored. This approach can be used to store information in the digital twin model. At the same time, the stored information can be processed and exported to be used to train the reinforcement learning model as described in this study. After the reinforcement learning model is trained and needs to be used for the real-world application, results from the application can also be saved to the digital twin model as shown in Fig. 14.

From the develop digital twin model, the data and information can be linked and stored in the digital twin model in real-time using the developed workflow presented in Fig. 8. Examples of data and information that can be linked and stored in the digital twin model are track location, track section, track geometry, track component defects, or maintenance activities. After that, the data stored in the digital twin model is linked to the developed reinforcement learning model. The reinforcement learning model will use the data extracted from the digital twin model to decide on the proper maintenance activities to perform in the following maintenance period. The suggested maintenance activities from the reinforcement learning model can be used to prepare the maintenance plan or maintenance report. Moreover, it can also be stored in the developed digital twin model using the developed workflow shown in Fig. 8. The findings conform to previous studies stating that the integration of digital twin and machine learning will promote the overall efficiency of project life not only at a specific stage of the project^28–30 ^ considering all risks and vulnerabilities^31–33^.

In addition, the developed approach to integrate digital twin and machine learning can also promote the building information modeling (BIM) maturity level. There are many definitions of BIM maturity levels depending on countries and organizations. One simple example is a definition by UNITED-BIM INC^34^. They classify the BIM maturity levels into four levels, from level 0 to level 3. Level 0 means BIM has not been applied and everything is paper-based. Level 1 means the 2D model is mainly used in the project while the 3D model is used for the conceptual purpose. Level 2 is the current goal for different countries because it promoted collaboration and each party has its own 3D model which is sharable among parties. The last one is Level 3 that the model is completely sharable and supports fully collaboration when every party works on the same single model. From the result of the study, it can be seen that the developed approach can fulfill the requirement of the BIM maturity level at least level 2 because it can support the collaboration between parties such as railway operators and maintenance operators. With good preparation, it can reach the goal of BIM maturity level 3 because every party can access the digital twin model to add information and apply information from the digital twin model completely. For example, inspection staff can collect field data and add information to the developed digital twin model. Then, data scientists can train the reinforcement learning model or use the collected field data as input to use the reinforcement learning model to prepare the predictive maintenance plan. After the maintenance is performed, the performed maintenance activities can be stored in the digital twin model for record and reinforcement learning model training. As mentioned, these activities are done by different parties who can share the same digital twin model. This is an example of the developed approach’s application to fulfill the goal of BIM maturity level 5.

## Conclusion

This study is the world's first to improve the railway infrastructure maintenance efficiency using deep reinforcement learning integrated with digital twin based on track geometry parameters and track component defects. the reinforcement learning technique used in this study is A2C. The reinforcement learning agent is trained to select the more suitable maintenance activities to perform in different situations. Rewards for the agent are based on maintenance costs and penalties when defects occur. Therefore, the agent has to learn how to perform maintenance activities properly. In other words, if the agent performs maintenance activities too many, defects may not be likely found but the maintenance cost will be high. The aim of the agent is to keep the maintenance cost as low as possible while track sections are free from defects. Different sources of data are used as a database to develop the environment for reinforcement learning. Sources of data are track geometry measurements, defect inspection reports, and maintenance records. It is worth noting that the data used in the study are real-world field data and the length of the section is 30 km collected between 2016 and 2019.

The developed reinforcement learning model can improve the maintenance efficiency according to the aim. In addition, it has the capability to handle the stochastic nature of the railway system when defects can take place even those track sections are well maintained. However, the agent of the reinforcement model can reduce a significant number of defects by up to 68% when the maintenance activities can be reduced by 20%. This will save a lot of maintenance costs, reduce possession time, and improve the reliability of the railway system. In addition, safety and passenger comfort will benefit from the improved track conditions. Therefore, it can be concluded that the developed reinforcement learning model has the potential and can fulfill the expected contribution of the study.

The new insights are highly beneficial for railway decision-making organizations to improve track inspection and maintenance schedules. They can use their data from the database to train the reinforcement learning model using the approach proposed in this study. Then, they can use the developed model to support decision-making or even drive decisions which is the ultimate goal of data-driven organizations. Some constraints can be added or adjusted in the environment to meet their conditions such as the limitation in resources in terms of budget, machine, or human resources. The degree of maintenance activities can also be added in action spaces to result in different states which will be interesting for the further stage of the study. In addition, the following inspection and measurement plan can rely on the current track conditions and performed maintenance activities as well. This will absolutely reflect the real-world application but increase the complication of the study as well. However, it is interesting and has the potential to develop reinforcement learning to imitate real-world situations as much as possible.

## Data Availability

The data that support the findings of this study are available from Brazilian Railway Authority but restrictions apply to the availability of these data, which were used under license for the current study, and so are not publicly available. Data are however available from the authors upon reasonable request and with permission of the Brazilian Railway Authority.
